# Knowledge, beliefs, and practices related to coronavirus disease 2019 (COVID-19) infection and vaccination in healthcare personnel working at nonacute care facilities

**DOI:** 10.1017/ice.2023.45

**Published:** 2023-10

**Authors:** Armaghan-e-Rehman Mansoor, Caroline A. O’Neil, David McDonald, Victoria J. Fraser, Hilary M. Babcock, Jennie H. Kwon, for the CDC Prevention Epicenters Program

**Affiliations:** 1 Division of Infectious Diseases, Department of Internal Medicine, Washington University in St. Louis, St. Louis, Missouri; 2 Department of Internal Medicine, Washington University in St. Louis, St. Louis, Missouri

## Abstract

**Objective:**

To characterize experiences, beliefs, and perceptions of risk related to coronavirus disease 2019 (COVID-19), infection prevention practices, and COVID-19 vaccination among healthcare personnel (HCP) at nonacute care facilities.

**Design:**

Anonymous survey.

**Setting:**

Three non–acute-care facilities in St. Louis, Missouri.

**Participants:**

In total, 156 HCP responded to the survey, for a 25.6% participation rate). Among them, 32% had direct patient-care roles.

**Methods:**

Anonymous surveys were distributed between April-May 2021. Data were collected on demographics, work experience, COVID-19 exposure, knowledge, and beliefs about infection prevention, personal protective equipment (PPE) use, COVID-19 vaccination, and the impact of COVID-19.

**Results:**

Nearly all respondents reported adequate knowledge of how to protect oneself from COVID-19 at work (97%) and had access to adequate PPE supplies (95%). Many HCP reported that wearing a mask or face shield made communication difficult (59%), that they had taken on additional responsibilities due to staff shortages (56%), and that their job became more stressful because of COVID-19 (53%). Moreover, 28% had considered quitting their job. Most respondents (78%) had received at least 1 dose of COVID-19 vaccine. Common reasons for vaccination were a desire to protect family and friends (84%) and a desire to stop the spread of COVID-19 (82%). Potential side effects and/or inadequate vaccine testing were cited as the most common concerns by unvaccinated HCP.

**Conclusions:**

A significant proportion of HCP reported increased stress and responsibilities at work due to COVID-19. The majority were vaccinated. Improving workplace policies related to mental health resources and sick leave, maintaining access to PPE, and ensuring clear communication of PPE requirements may improve workplace stress and burnout.

The coronavirus disease 2019 (COVID-19) pandemic has had a disproportionate impact on nonacute care facilities. Some of the highest rates of infection and mortality have occurred among residents and healthcare personnel (HCP) at these sites.^1–3^ Staff in nonacute care facilities may also work at multiple facilities, which can amplify the spread of respiratory viruses across these settings.^4^ Implementing infection control measures in long-term care and nonacute care facilities can also be challenging. For example, memory care facilities may face challenges with isolation practices and restricting patient movement.^5^ In addition, acute care facilities generally have access to healthcare personnel trained in infection prevention, which may not be the case in nonacute care settings.^6^

HCP in nonacute care settings can also be vulnerable to changes in availability of PPE, and they may have limited training on appropriate PPE use.^7^ Rapid changes in the availability of COVID-19 diagnostics, transmission and quarantine protocols, treatment considerations and vaccine availability create additional challenges for HCP.^8,9^ Considering these challenges, there is a high concern that the ongoing pandemic will affect the physical and mental well-being of HCP in nonacute care settings.^10,11^

Recent literature shows increased psychological stressors, anxiety, sleep disturbances, increased workload and burnout among HCP in acute care settings during the COVID-19 pandemic.^11–14^ Surveys of HCP at acute care facilities during the pandemic have also shown significant heterogeneity in the knowledge and attitudes toward COVID-19 transmission, infection prevention, and COVID-19 vaccination.^15–17^ Prior studies of respiratory virus transmission in a single LTCF have revealed knowledge gaps regarding recommended infection prevention practices.^18^ However, limited information is available on the experiences, attitudes, and infection prevention practices related to COVID-19, as well as the impact of the pandemic on HCP working in nonacute care facilities. Therefore, we assessed the experience, knowledge, beliefs, and perceptions of risk related to COVID-19, infection prevention practices, and COVID-19 vaccination among HCP employed in 3 nonacute care facilities.

## Methods

In April and May 2021, an anonymous survey was distributed to HCP at 3 nonacute care facilities in the St. Louis, Missouri, metropolitan area. To include a range of nonacute care settings, 1 skilled nursing facility, 1 memory care facility, and 1 postacute rehabilitation facility were selected as the sites for this survey; these facilities had inpatient capacities of 56, 125, and 96 beds, respectively. The total number of staff employed at the nonacute care facilities at the time of survey completion was estimated to be 620. The survey included 83 total questions about demographics (age, sex, race, ethnicity), experience working in healthcare, job role, frequency of contact with patients with COVID-19, and COVID-19 history and exposures. Respondents were also asked to respond to a series of statements about COVID-19 beliefs and knowledge, SARS-CoV-2 vaccination, COVID-19 policies and practices at their facility, behaviors at work and outside work, fears related to COVID-19 and their job, and the impact of COVID-19 on the respondent. The survey was based on a prior study on respiratory infections in long-term care facilities.^18^

A secure link to complete the survey electronically was distributed to all facility employees via email. Paper copies of the survey were also placed in staff work areas at each facility and could be placed in sealed envelopes in a deposit box. Respondents reviewed a consent information sheet prior to completing the survey. Completion of the survey was considered consent for study participation. Respondents were allowed to skip any questions that they preferred not to answer.

Survey data were collected and managed using REDCap electronic data capture tools hosted at Washington University in St. Louis.^19,20^ The analysis was completed using SPSS Statistics version 27 software (IBM, Armonk, NY). Responses to each survey question were summarized and then stratified by facility and extent of HCP contact with patients with COVID-19. Using Mann-Whitney *U* tests, descriptive statistics were calculated for group comparisons of survey responses from HCP who had more versus less contact with COVID-19 patients.

The study protocol was approved by the Washington University Human Research Protection Office (IRB no. 202102194) with a waiver of consent for recruitment and a waiver of documentation of consent.

## Results

### Attitudes, beliefs, and practices related to COVID-19 infection prevention and PPE use

In total, 156 HCP responded to the survey, with most surveys completed on paper (n = 104, 66.7%). Respondents were evenly divided among the 3 facilities, and one-third of HCP reported healthcare experience of 15 or more years. The demographics and workplace roles for the survey respondents are noted in Table 1. The overall survey participation rate was 25.2%: 42.5% at the skilled nursing facility, 23.5% at the memory care facility, and 19.0% at the postacute rehabilitation facility. Furthermore, 87.8% of HCP reported having been tested for COVID-19 at some time in the past, and 20.5% reported having previously tested positive for COVID-19. Nearly one-quarter of respondents (23.1%) reported having someone in their household who tested positive for COVID-19; 46.8% reported knowing someone outside work who had been hospitalized for COVID-19; and 41.7% reported knowing someone outside work who had died of COVID-19.

**Table 1.** Demographics and Job Roles of Surveyed Healthcare Personnel

Variable	No. (%) (N = 156)
**Sex**	
Female	122 (78.2)
Male	22 (14.1)
Nonbinary	2 (1.3)
Prefer not to answer or did not answer	10 (6.4)
**Age**	
18–35 y	59 (37.8)
36–55 y	58 (37.2)
≥56 y	31 (19.9)
Age missing	8 (5.1)
**Race and ethnicity**	
White/Caucasian	81 (51.9)
Black/African American	54 (34.6)
Asian	3 (1.9)
Multiracial	5 (3.2)
Other	10 (6.4)
Race missing	3 (1.9)
Hispanic or Latino	6 (3.8)
Not Hispanic or Latino	130 (83.3)
Ethnicity missing	20 (12.8%)
**Experience working in healthcare**	
0–4 y	37 (23.7)
5–9 y	34 (21.8)
10–14 y	29 (18.6)
≥15 y	53 (34.0)
Missing	3 (1.9)
**Job role**	
Activities	10 (6.4)
Administration	23 (14.7)
Dining services/dietician	13 (8.3)
Environmental services/facilities	10 (6.4)
Nurses assistant/patient-care technician	25 (16.0)
Nurse	24 (15.4)
Advance practice nurse, NP, or PA	2 (1.3)
Pharmacist	2 (1.3)
Therapy (PT, OT, ST, RT)	19 (12.2)
Social worker/case manager	7 (4.5)
Not specified	21 (13.5)

Note. NP, nurse practitioner; PA, physician assistant; PT, physical therapist; OT, occupational therapist; ST, speech therapist; RT, respiratory therapist.

Overall, 78.7% of survey respondents agreed that COVID-19 was a serious problem in the community, and 29.7% agreed that COVID-19 was a serious problem at their facility. Also, 97.5% of respondents knew that asymptomatic COVID-19 infection could occur, and 81.9% stated that wearing a face mask could help protect them from COVID-19. However, 58.7% agreed that wearing a mask made it more difficult to communicate with others. Furthermore, 81.3% of respondents reported having received adequate training on how to put on and remove PPE. Although 90.3% of HCP stated that PPE was readily available at their facility at the time of the survey, 27.7% stated that they had had to reuse PPE in the past due to supply shortages. Also, 31.0% stated that it was difficult to keep up with changing rules and recommendations regarding PPE use during the pandemic.

### Differences in COVID-19–related practices by degree of contact with patients known or suspected to have COVID-19

In total, 145 HCP provided information about how frequently they had contact with patients who were known or suspected to have COVID-19 while at work, 58 (40.0%) reported contact at least a few days per week. Survey responses were stratified by the extent of HCP contact with known or suspected patients with COVID-19 and are detailed in Table 2. Notably, a higher proportion of respondents with less contact with patients with COVID-19 agreed that they had inadequate PPE due to supply shortages, with the *P* value approaching significance (4.6% vs 10.3%; *P =* .06).

**Table 2.** Respondent COVID-19 Beliefs, Knowledge, PPE Use, Social Distancing Practices, Fears, and Impact, Stratified by Extent of Contact with Patients with COVID-19

Statement	Less Contact With COVID Patients (Once a Week or Less, N = 87), No. (%)			More Contact With COVID Patients (A Few Days a Week or More, N = 58), No. (%)			
	Agree	Neutral	Disagree	Agree	Neutral	Disagree	*P* Value
**Beliefs**							
COVID-19 is a serious problem in our community	68 (78.2)	10 (11.5)	7 (8.0)	47 (81.0)	7 (12.1)	2 (3.4)	.50
COVID-19 is a serious problem at my facility	25 (28.7)	22 (25.3)	38 (43.7)	19 (32.8)	20 (34.5)	18 (31.0)	.22
Wearing a face mask helps protect me from getting COVID-19	70 (80.5)	12 (13.8)	3 (3.4)	49 (84.5)	1 (1.7)	8 (13.8)	.97
Wearing a mask and/or face shield makes it hard to communicate with others	50 (57.5)	20 (23.0)	15 (17.2)	36 (62.1)	10 (17.2)	10 (17.2)	.61
**Knowledge**							
I know how to protect myself from COVID-19 while I am at work	84 (96.6)	1 (1.1)	1 (1.1)	56 (96.6)	0	2 (3.4)	.68
I know how to protect myself from COVID-19 while I am out in public	83 (95.4)	2 (2.3)	1 (1.1)	56 (96.6)	0	2 (3.4)	.99
People can have COVID-19 without having any symptoms	77 (88.5)	8 (9.2)	1 (1.1)	55 (94.8)	2 (3.4)	1 (1.7)	.27
COVID-19 spreads easily from person to person	74 (85.1)	10 (11.5)	1 (1.1)	48 (82.8)	7 (12.1)	2 (3.4)	.60
I have received adequate training on how to put on and remove PPE	70 (80.5)	9 (10.3)	5 (5.7)	48 (82.8)	4 (6.9)	4 (6.9)	.72
**Personal protective equipment (PPE)**							
The PPE that I need to protect myself from COVID-19 is easily available to me at my facility	79 (90.8)	4 (4.6)	1 (1.1)	53 (91.4)	0	3 (5.2)	.87
Because of supply shortages, I have had to re-use PPE	26 (29.9)	9 (10.3)	48 (55.2)	16 (27.6)	9 (15.5)	31 (53.4)	.95
Because of supply shortages, I have had to use inadequate PPE while caring for patients with COVID-19	4 (4.6)	4 (4.6)	73 (83.9)	6 (10.3)	6 (10.3)	44 (75.9)	.06
I have found it difficult to keep up with changes in PPE rules and requirements during the COVID-19 pandemic	26 (29.9)	17 (19.5)	38 (43.7)	21 (36.2)	13 (22.4)	20 (34.5)	.28
**Social distancing**							
My facility has provided space where I can be distanced from others while eating and/or taking breaks	63 (72.4)	13 (14.9)	5 (5.7)	46 (79.3)	3 (5.2)	5 (10.3)	.53
I practice social distancing when I am at work and not involved in immediate patient care	73 (83.9)	8 (9.2)	2 (2.3)	51 (87.9)	3 (5.2)	2 (3.4)	.59
My coworkers practice social distancing when they are at work and not involved in patient care	58 (66.7)	19 (21.8)	6 (6.9)	46 (79.3)	7 (12.1)	3 (5.2)	.12
**Fears**							
I am afraid of getting COVID-19 at work	27 (31.0)	27 (31.0)	27 (31.0)	20 (34.5)	10 (17.2)	23 (39.7)	.70
I am afraid of getting COVID-19 somewhere other than work	40 (46.0)	24 (27.6)	18 (20.7)	28 (48.3)	5 (8.6)	20 (34.5)	.60
I have been afraid to go to work because of COVID-19	19 (21.8)	18 (20.7)	44 (50.6)	6 (10.3)	9 (15.5)	36 (62.1)	.49
**Impact**							
My facility has provided adequate mental health support for staff during the pandemic	21 (24.1)	38 (43.7)	23 (26.4)	15 (25.9)	23 (39.7)	17 (29.3)	.92
I have had to take on extra responsibilities at work due to staff shortages related to COVID	43 (49.4)	11 (12.6)	27 (31.0)	37 (63.8)	10 (17.2)	7 (12.1)	.027
I have considered quitting my job because of COVID-19	13 (14.9)	12 (13.8)	56 (64.4)	5 (8.6)	9 (15.5)	38 (65.5)	.52
I have considered leaving the health care profession or retiring early because of COVID-19	9 (10.3)	13 (14.9)	58 (66.7)	4 (6.9)	7 (12.1)	42 (72.4)	.37
I have felt discriminated against by the public because I am a healthcare professional	11 (12.6)	29 (20.7)	50 (57.5)	6 (10.3)	10 (17.2)	37 (63.8)	.45

### Impact of COVID-19 on HCP at nonacute care facilities

Only 56.1% of respondents reported that their facility made it easy for them to stay home when they were sick, and only 25.2% stated that adequate mental health support had been provided to staff during the pandemic. Many HCP (56.1%) reported having taken on extra responsibilities at work due to staff shortages related to COVID-19, and 52.9% stated that their job had become more stressful because of COVID-19. Half of respondents (50.3%) stated that they felt they were at higher risk for getting COVID-19 because they worked at a healthcare facility, and 18.1% stated that they had been afraid to go to work because of COVID-19. Perhaps because of these factors, 13.5% of survey respondents stated that they had contemplated quitting their job because of COVID-19. HCP with more contact with patients with COVID-19 were more likely to agree with the statement that they had taken on additional responsibilities at work due to staff shortages (63.8% vs 49.4%; *P =* .027).

### Attitudes and beliefs regarding COVID-19 vaccination

When asked about COVID-19 vaccination, 78.1% of respondents reported having received at least 1 dose of COVID-19 vaccine, and 71.6% reported having completed a primary vaccine series. Among vaccinated HCP, the most common reasons given for having decided to be vaccinated were to protect family and friends (84.3%), to help stop the spread of COVID-19 (81.8%), to protect themselves (78.5%), and to protect their patients (73.6%). Among the 34 HCP who reported not having received a COVID-19 vaccine, 9.7% stated that they did not intend to be vaccinated. Among unvaccinated HCP who stated that they did not intend to be vaccinated, the most common reasons given for this decision were concern about vaccine side effects (52.9%), concern that the vaccine had not been tested well enough (50.0%), and concern about how quickly the vaccine was developed (47.1%).

## Discussion

Optimal infection prevention in nonacute care facilities has been a unique public health challenge during the COVID-19 pandemic. Disproportionately high rates of COVID-19 spread among patients and/or residents and HCP in nonacute care facilities have been reported.^1–3^ Nevertheless, studies evaluating the impact of the pandemic on HCP have primarily focused on acute care facilities.^10–13^ In this study, we addressed this knowledge gap by focusing on practices, knowledge, and attitudes regarding COVID-19 among HCP working in 3 types of nonacute care facilities.

Most HCP responding to the survey reported accurate knowledge about COVID-19 transmission and were confident in their ability to protect themselves from infection. However, 31% of HCP reported difficulty keeping up with changing PPE rules and requirements as the pandemic evolved. Implementing effective and concise communication regarding changes in PPE, both at institutional or regional levels, may help reduce potential confusion among HCP. More than half of the HCP who responded to the survey also reported that wearing a mask made communication difficult. This difficulty has been reported as a concern by both the general public and HCP in other studies, especially among hearing-impaired individuals, and strategies such as clear masks have had limited success and raise concerns about PPE effectiveness.^21,22^

Half of the survey respondents felt they were at greater risk for becoming infected because they worked at a healthcare facility, and nearly 20% stated that they had been afraid to go to work. Notably, however, it remains unclear whether HCP who had more frequent contact with patients with COVID-19 were more likely to acquire infection. Studies have shown that the incidence of COVID-19 infection among HCP, regardless of the degree of contact with patients with COVID-19 at their place of work, more closely aligns with the rate of COVID-19 transmission in the general population.^23,24^

Several studies have reported high rates of burnout and stress among HCP working at acute care facilities during the COVID-19 pandemic.^25–28^ Our study revealed a similar pattern among HCP working in nonacute care settings. More than half of the HCP who responded to this survey reported additional work responsibilities and stress due to the pandemic. A notable percentage of HCP (13.5%) had considered leaving their job because of COVID-19. This finding is significant and underscores the need for strategies to mitigate burnout among HCP and to improve retention at LCTFs. Notably, only 25% of the HCP who responded to our survey stated that their facility had provided adequate mental health support to staff during the pandemic, and only half stated that it was easy for them to stay home from work when they were sick. A major strength of this study was including HCP with different job roles and varying levels of contact with patients with COVID-19. This allowed us to evaluate differences in practices and attitudes between HCP who had more versus less contact with patients with COVID-19. Although most beliefs and practices were similar among the 2 cohorts, HCP with more contact with patients with COVID-19 were more likely to agree that they had taken on additional responsibilities due to staff shortages (63.8% vs 49.4%; *P =* .027), compounding the concern for the impact of the pandemic on HCP taking care of patients with COVID-19.

Although >75% of the HCP who responded to the survey had received at least 1 dose of COVID-19 vaccine, several had not yet been vaccinated, and some did not intend to be vaccinated. Reasons that HCP provided for their vaccine hesitancy were similar to those reported in a study evaluating HCP at acute care facilities^29^ and included concerns about side effects, rapid vaccine deployment, and inadequate vaccine testing. Since this study was completed, COVID-19 vaccine formulations have received full approval from the Food and Drug Administration (FDA), and booster doses have been recommended by public health guidelines. Future studies could re-examine the prevalence and causes of vaccine hesitancy in this cohort following these changes. Notably, as the pandemic has evolved, studies have shown that societal attitudes toward pandemic-related rules and regulation have undergone a paradigm shift and continue to move away from compliance due to fear alone; these attitudes appear to have become increasingly nuanced.^30,31^

This study had several limitations. Guidance regarding PPE use, vaccination requirements, isolation practices have gone through multiple iterations during the COVID-19 pandemic and the availability of resources (eg, PPE, COVID-19 testing and vaccination) have also been quite variable, especially early in the pandemic. Although this survey provides insights into the attitudes and knowledge regarding COVID-19 among HCP working in nonacute care settings at the time of the survey, practices have changed since this time and are likely to further evolve. Future research might focus on re-evaluating the knowledge and beliefs of HCP in nonacute care settings as the healthcare guidelines regarding infection prevention, PPE use, and mandatory vaccination policies are updated. These data were acquired via an anonymous survey to promote honest responses without concern for replies being tracked to an individual. As such, all data were self-reported and did allow for a strategy to ensure nonduplication of responses. Additionally, given the survey participation rate of 25.2%, the HCP who chose to respond may not be representative of all staff who worked at the participating facilities. The results of this survey may also not be generalizable to other nonacute care facilities.

Despite these limitations, these findings provide important insights into the experience, knowledge, beliefs, and perceptions of risk related to COVID-19, infection prevention practices, and COVID-19 vaccination among HCP employed in nonacute care settings. To our knowledge, this is the first in-depth survey that has focused on the impact of COVID-19 on HCP in nonacute care facilities. Additional studies are also warranted to investigate differences in such perceptions and practices between various types of nonacute care facilities. Findings from this survey and future studies can be used to improve infection prevention practices, to design strategies to increase vaccine uptake, to reduce burnout among HCP and improve workforce retention, and to help design interventions to improve occupational health and safety for HCP working at nonacute care facilities at regional and national levels.

## References

[1] McMichael TM , Clark S , Pogosjans S , et al. COVID-19 in a long-term care facility— King County, Washington, February 27–March 9, 2020. Morb Mortal Wkly Rep 2020;69:339–342. 10.15585/mmwr.mm6912e1 PMC7725515 32214083

[2] Roxby AC , Greninger AL , Hatfield KM , et al. Outbreak investigation of COVID-19 among residents and staff of an independent and assisted living community for older adults in Seattle, Washington. JAMA Intern Med 2020;180:1101–1105. 32437547 10.1001/jamainternmed.2020.2233 PMC7292007

[3] Kluytmans-van den Bergh MFQ , Buiting AGM , Pas SD , et al. Prevalence and clinical presentation of healthcare workers with symptoms of coronavirus disease 2019 in 2 Dutch hospitals during an early phase of the pandemic. JAMA Network Open 2020;3:e209673. 32437576 10.1001/jamanetworkopen.2020.9673 PMC7243090

[4] Van Houtven CH , DePasquale N , Coe NB. Essential long-term care workers commonly hold second jobs and double- or triple-duty caregiving roles. J Am Geriatr Soc 2020;68:1657–1660. 32338767 10.1111/jgs.16509 PMC7267626

[5] Velayudhan L , Aarsland D , Ballard C. Mental health of people living with dementia in care homes during COVID-19 pandemic. Int Psychogeriatr 2020;32:1253–1254. 32487278 10.1017/S1041610220001088 PMC7302947

[6] Hori H , Fukuchi T , Sanui M , Moriya T , Sugawara H. Comprehensive infection control measures prevent hospital-acquired severe acute respiratory syndrome coronavirus 2 infection: a single-center prospective cohort study and seroprevalence survey. PLoS One 2021;16:e0257513. 34634076 10.1371/journal.pone.0257513 PMC8504754

[7] Abbasi J. “Abandoned” nursing homes continue to face critical supply and staff shortages as COVID-19 toll has mounted. JAMA 2020;324:123–125. 32525535 10.1001/jama.2020.10419

[8] Ness MM , Saylor J , Di Fusco LA , Evans K. Healthcare providers’ challenges during the coronavirus disease (COVID-19) pandemic: a qualitative approach. Nurs Health Sci 2021;23:389–397. 33580590 10.1111/nhs.12820 PMC8012981

[9] Razu SR , Yasmin T , Arif TB , et al. Challenges faced by healthcare professionals during the COVID-19 pandemic: a qualitative inquiry from Bangladesh. Front Public Health 2021;9:647315. 34447734 10.3389/fpubh.2021.647315 PMC8383315

[10] Chew NWS , Lee GKH , Tan BYQ , et al. A multinational, multicentre study on the psychological outcomes and associated physical symptoms amongst healthcare workers during COVID-19 outbreak. Brain Behav Immun 2020;88:559–565. 32330593 10.1016/j.bbi.2020.04.049 PMC7172854

[11] Felice C , Di Tanna GL , Zanus G , Grossi U. Impact of COVID-19 outbreak on healthcare workers in italy: results from a national e-survey. J Community Health 2020;45:675–683. 32440724 10.1007/s10900-020-00845-5 PMC7242177

[12] Pappa S , Ntella V , Giannakas T , Giannakoulis VG , Papoutsi E , Katsaounou P. Prevalence of depression, anxiety, and insomnia among healthcare workers during the COVID-19 pandemic: a systematic review and meta-analysis. Brain Behav Immun 2020;88:901–907. 32437915 10.1016/j.bbi.2020.05.026 PMC7206431

[13] Wu Y , Wang J , Luo C , et al. A comparison of burnout frequency among oncology physicians and nurses working on the frontline and usual wards during the COVID-19 epidemic in Wuhan, China. J Pain Symptom Manage 2020;60:e60–e65. 32283221 10.1016/j.jpainsymman.2020.04.008 PMC7151285

[14] Liu CY , Yang YZ , Zhang XM , et al. The prevalence and influencing factors in anxiety in medical workers fighting COVID-19 in China: a cross-sectional survey. Epidemiol Infect 2020;148:e98. 32430088 10.1017/S0950268820001107 PMC7251286

[15] Maude RR , Jongdeepaisal M , Skuntaniyom S , et al. Improving knowledge, attitudes and practice to prevent COVID-19 transmission in healthcare workers and the public in Thailand. BMC Public Health 2021;21:749. 33865342 10.1186/s12889-021-10768-y PMC8053080

[16] Nguyen KH , Yankey D , Coy KC , et al. COVID-19 vaccination coverage, intent, knowledge, attitudes, and beliefs among essential workers, United States. Emerg Infect Dis 2021;27:2908–2913. 34586060 10.3201/eid2711.211557 PMC8544962

[17] Nguyen KH , Irvine S , Chung M , et al. Prevalence of previous COVID-19 infection, COVID-19 vaccination receipt, and intent to vaccinate among the US workforce. Public Health Rep 2022;137:755–763. 35403489 10.1177/00333549221085238 PMC9066271

[18] O’Neil CA , Kim L , Prill MM , et al. Preventing respiratory viral transmission in long-term care: knowledge, attitudes, and practices of healthcare personnel. Infect Control Hosp Epidemiol 2017;38:1449–1456. 29173225 10.1017/ice.2017.232

[19] Harris PA , Taylor R , Thielke R , Payne J , Gonzalez N , Conde JG. Research electronic data capture (REDCap)—a metadata-driven methodology and workflow process for providing translational research informatics support. J Biomed Inform 2009;42:377–381. 18929686 10.1016/j.jbi.2008.08.010 PMC2700030

[20] Harris PA , Taylor R , Minor BL , et al. The REDCap consortium: building an international community of software platform partners. J Biomed Inform 2019;95:103208. 31078660 10.1016/j.jbi.2019.103208 PMC7254481

[21] Chu JN , Collins JE , Chen TT , et al. Patient and healthcare worker perceptions of communication and ability to identify emotion when wearing standard and transparent masks. JAMA Netw Open 2021;4:e2135386. 34807257 10.1001/jamanetworkopen.2021.35386 PMC8609412

[22] Wong CK , Yip BH , Mercer S , et al. Effect of face masks on empathy and relational continuity: a randomised controlled trial in primary care. BMC Fam Pract 2013;14:200. 24364989 10.1186/1471-2296-14-200 PMC3879648

[23] Treibel TA , Manisty C , Burton M , et al. COVID-19: PCR screening of asymptomatic healthcare workers at London hospital. Lancet 2020;395:1608–1610. 32401714 10.1016/S0140-6736(20)31100-4 PMC7206444

[24] Baker JM , Nelson KN , Overton E , et al. Quantification of occupational and community risk factors for SARS-CoV-2 seropositivity among healthcare workers in a large US healthcare system. Ann Intern Med 2021;174:649–654. 33513035 10.7326/M20-7145 PMC7877798

[25] Lai J , Ma S , Wang Y , et al. Factors associated with mental health outcomes among healthcare workers exposed to coronavirus disease 2019. JAMA Netw Open 2020;3:e203976. 32202646 10.1001/jamanetworkopen.2020.3976 PMC7090843

[26] Khanagar SB , Al-Ehaideb A , Vishwanathaiah S , Maganur PC , Varadarajan S , Patil S. Depression, anxiety, and psychological distress among healthcare providers during the outbreak of the life-threatening coronavirus disease (COVID-19). J Contemp Dent Pract 2020;21:471–472. 32690824

[27] Murat M , Köse S , Savaşer S. Determination of stress, depression and burnout levels of front-line nurses during the COVID-19 pandemic. Int J Ment Health Nurs 2021;30:533–543. 33222350 10.1111/inm.12818 PMC7753629

[28] Salari N , Khazaie H , Hosseinian-Far A , et al. The prevalence of stress, anxiety and depression within frontline healthcare workers caring for COVID-19 patients: a systematic review and meta-regression. Hum Resour Health 2020;18:100. 33334335 10.1186/s12960-020-00544-1 PMC7745176

[29] Browne SK , Feemster KA , Shen AK , et al. Coronavirus disease 2019 (COVID-19) vaccine hesitancy among physicians, physician assistants, nurse practitioners, and nurses in two academic hospitals in Philadelphia. Infect Control Hosp Epidemiol 2022;43:1424–1432. 34538290 10.1017/ice.2021.410 PMC8503076

[30] Six F , de Vadder S , Glavina M , Verhoest K , Pepermans K. What drives compliance with COVID-19 measures over time? Explaining changing impacts with Goal Framing Theory. Regul Gov 2021. doi: 10.1111/rego.12440. PMC8661714 34909051

[31] Nivette A , Ribeaud D , Murray A , et al. Noncompliance with COVID-19-related public health measures among young adults in Switzerland: insights from a longitudinal cohort study. Soc Sci Med 2021;268:113370. 32980677 10.1016/j.socscimed.2020.113370 PMC7493799

